# A Case Report of Grave’s Disease Presenting With an Episode of Restless Legs Syndrome

**DOI:** 10.7759/cureus.57354

**Published:** 2024-03-31

**Authors:** Kazuki Miyaue, Hiroki Isono

**Affiliations:** 1 Department of General Medicine, HITO Medical Center, Ehime, JPN

**Keywords:** secondary rls, sleep medicine, neurology, grave's disease, restless legs syndrome

## Abstract

This case report explores a rare presentation of restless legs syndrome (RLS) in a 59-year-old female with a history of Graves' disease (GD), highlighting the diagnostic challenges and the importance of considering thyroid dysfunctions in RLS. The patient, previously diagnosed and treated for GD, presented with acute nocturnal discomfort in her lower limbs, along with symptoms of fatigue, weight loss, and palpitations. Physical examinations and thyroid function tests indicated a recurrence of GD. Her RLS symptoms notably resolved following the treatment of GD with methimazole, pointing towards secondary RLS induced by GD. This case emphasizes the necessity of a comprehensive diagnostic approach in RLS, particularly in differentiating it from mimicking conditions including leg cramps, arthritis, peripheral neuropathy, and drug-induced akathisia, and identifying underlying etiologies like thyroid dysfunctions. The resolution of RLS symptoms after GD treatment underlines the potential link between thyroid dysfunctions, iron metabolism, and the dopaminergic system in RLS pathophysiology.

## Introduction

Restless legs syndrome (RLS) is a neurological condition characterized by an insatiable urge to move the limbs, frequently accompanied by disagreeable sensations [1]. These symptoms predominantly manifest nocturnally, exacerbating during periods of rest and alleviating with movement [1]. Moreover, epidemiological studies suggest the prevalence of clinically significant RLS in approximately 2-3% of the adult population [2,3]. RLS is said to be associated with genetic factors, as suggested by research showing that early-onset RLS tends to follow a genetic pattern involving one dominant gene, inherited in an autosomal-dominant manner [4]. Moreover, it is linked to the dopaminergic system and iron levels, along with predisposing conditions, but much remains unknown [1,4].

Graves' disease (GD), which commonly presents with weight loss, fatigue, heat intolerance, tremors, and palpitations, is an autoimmune thyroid disorder influenced by genetic and environmental factors, adversely affecting the quality of life and increasing mortality risk [5].

Despite the scarcity of prior cases linking RLS and thyroid diseases, we present a unique case fulfilling the diagnostic criteria of RLS that was eventually diagnosed with GD.

## Case presentation

A 59-year-old woman presented with a primary complaint of persistent discomfort in both lower limbs. This discomfort, which was initiated approximately three weeks prior to her visit, notably exacerbated nocturnally. Furthermore, the patient’s condition escalated approximately one week before her presentation, with an onset of fatigue, weight loss, and palpitations. Merely two days before seeking medical care, her lower extremity symptoms intensified during the night, manifesting as an inability to remain still, albeit temporarily alleviated by leg movement. She had not had any similar episodes before. 

The patient's medical history revealed a pivotal diagnosis of GD at the age of 47, initially managed with methimazole for two years and achieving remission. However, a relapse occurred at the age of 52, necessitating the recommencement of methimazole, which was subsequently discontinued after a year (she did not experience any leg discomfort during the first and second episodes). She did not have diabetes. She did not have a family history of RLS. The patient’s oral medication regimen included olmesartan, solifenacin, and magnesium oxide.

Upon physical examination, her vital signs were as follows: body temperature, 37.0 ℃; pulse, 92 beats per min; blood pressure, 120/64 mmHg; oxygen saturation, 98% in room air; and respiratory rate, 18 per min. Additionally, head and neck examination revealed no palpebral conjunctival pallor, proptosis, thyroid enlargement or tenderness, or cervical lymphadenopathy. Chest assessment indicated symmetrical breathing sounds, absence of crackles or wheezes, and regular heart sounds without murmurs. Abdominal examination showed a flat, soft, and non-tender abdomen. Extremities exhibited no edema, skin rash, or tremors.

Given the patient’s past medical history, thyroid function tests were conducted, revealing an aberrant thyroid-stimulating hormone (TSH) level (<0.05 μIU/ml) and elevated fT4 level (>7.77 ng/ml) (Table 1). Iron study yielded high ferritin levels at 385.6 ng/dl and comprehensive blood count, liver function test, and renal function test were normal. Notably, the TSH receptor antibody level was substantially elevated at 3.3 IU/L, leading to the diagnosis of a GD recurrence.

**Table 1:** Laboratory tests Hb: hemoglobin, PLT: platelet, ALB: albumin, T-Bil: total bilirubin, AST: aspartate aminotransferase, ALT: alanine aminotransferase, LDH: lactate dehydrogenase, CPK: creatine phosphokinase, BUN: blood urea nitrogen,  Na: sodium, K: potassium, Cl: chloride,  Ca: calcium, Glu: glucose, HbA1c: hemoglobin A1c, Fe: iron, TIBC: total iron binding capacity, UIBC: unsaturated iron binding capacity, TSH: thyroid stimulating hormone, FT4: free thyroxine, TSHR-Ab thyroid stimulating hormone receptor antibody

Laboratory tests	Results	Reference range
Leukocyte	4,290/µl	3,300-8,600/µl
Hb	12.7g/dl	11.6-14.8g/dl
PLT	210,000/µl	158,000-348,000/µl
ALB	4.2g/dl	3.8-5.3g/dl
T-Bil	0.3mg/dl	0.3-1.2mg/dl
AST	35U/l	10-40U/l
ALT	49U/l	5-40U/l
LDH	158U/l	124-222U/l
CPK	55U/l	45-163U/l
BUN	15.5mg/dl	8.0-22.0mg/dl
Creatinine	0.72mg/dl	0.46-0.79mg/dl
Na	140mmol/l	136-147mmol/l
K	4.5mmol/l	3.6-5.0mmol/l
Cl	104mmol/l	98-109mmol/l
Ca	9.6mg/dl	8.5-10.2mg/dl
Random Glu	141mg/dl	70-109mg/dl
HbA1c	5.90%	4.6-6.2%
Fe	71µg/dl	48-154µg/dl
TIBC	236µg/dl	246-410µg/dl
UIBC	165µg/dl	108-325µg/dl
Ferritin	385.6ng/dl	3.6-114ng/dl
TSH	<0.005µIU/ml	0.61-4.23µIU/ml
FT4	>7.77ng/dl	0.75-1.45ng/dl
TSHR-Ab	3.3IU/l	<1.0IU/l

Treatment commenced with methimazole at 30 mg/day. Remarkably, one month after treatment initiation, the patient’s lower limb symptoms resolved and thyroid function normalized. Methimazole dosage was gradually tapered, and the patient continued a maintenance dose of 5 mg/day nine months after starting treatment.

## Discussion

In the field of neurology, the case discussed here highlights two pivotal findings. First, the diagnostic process for RLS necessitates a meticulous exclusion of mimicking conditions and underlying etiologies. Second, our case underscores the quintessential role of thyroid dysfunctions, particularly in their ability to mimic RLS, and the necessity of considering thyroid dysfunctions when suspecting RLS. 

The revised diagnostic criteria of RLS (an urge to move one's legs with feelings of discomfort, the urge starting or worsening during periods of rest or inactivity, the urge relieved by movement, the urge worsening in the evening or at night, and ruling out other mimicking conditions) emphasize the importance of differentiating RLS from other conditions, and improve the specificity of the criteria [6]. In our case, we carefully ruled out the mimicking conditions mentioned in the criteria such as leg cramps, arthritis, peripheral neuropathy, and drug-induced akathisia [6]. It is also important to exclude secondary RLS, an underlying condition that causes symptoms that mimic primary RLS. Common secondary RLS includes iron deficiency, end-stage kidney disease, and pregnancy [7,8]. In contrast to primary RLS, secondary RLS develops more rapidly, and it can remit when the underlying conditions are resolved [7,9]. In our case, the symptoms acutely developed; thus, we suspected secondary RLS.

In concordance with our second finding, the role of thyroid dysfunctions in mimicking RLS symptoms is strikingly evident in our case. Some reviews suggest checking for thyroid dysfunctions when suspecting secondary RLS; however, studies supporting the description are lacking [7,8,10]. A study from Brazil revealed that 20% of patients with GD, who did not have RLS symptoms prior to GD, developed definite RLS or RLS-like symptoms, that lacked the definite RLS circadian characteristics during GD episodes [11]. Moreover, at least 70% of the patients reported that the RLS symptoms resolved when they turned euthyroid [11]. In our case, the symptoms fully resolved once methimazole administration began and GD remitted, which implied that GD caused the symptoms. A hypothesis of a potential link between RLS and hyperthyroidism is as follows [12]: dopamine, a key neurotransmitter in the regulation of motor activity and pleasure, also influences the secretion of TSH and, consequently, thyroid hormone levels. The regulation of these hormones is implicated in the modulation of RLS symptoms, with dopaminergic agonists providing relief and dopaminergic antagonists worsening the condition. This intricate balance suggests that thyroid hormones, when unopposed by adequate dopaminergic activity, may exacerbate or precipitate RLS symptoms, underlining a potential pathophysiological link between hyperthyroidism and RLS.

Another study reported that patients with thyroid disease were more likely to develop RLS [13]. Especially, the risk of RLS in patients with Hashimoto’s disease was 2.56 times higher than that in the control group [13]. Additionally, the occurrence of RLS is notably more common in individuals with hypothyroidism compared to those without it, and the incidence of hypothyroidism is substantially greater in individuals who have RLS than in those who do not [14]. The potential link between RLS and hypothyroidism encompasses complex interactions involving iron metabolism, the dopaminergic system, and neuroendocrine pathways [14]. The details are as follows: both conditions share associations with iron deficiency and anemia, suggesting that disturbances in iron metabolism could be a common pathway. Treatment of iron deficiency leads to changes in thyroid hormone levels, hinting at a cellular mechanism where thyroid function interacts with the iron system, potentially influencing RLS development. Furthermore, the melanocortin hormones, related to clinical features of RLS, hint at a direct association beyond iron metabolism. These hormones, influenced by dopaminergic activity, exhibit properties such as causing locomotion and pain sensitization in models, mirroring RLS symptoms. Their production is regulated by pro-opiomelanocortin (POMC) neurons, which are affected by thyroid hormone activity, suggesting a link through neuroendocrine regulation. Moreover, It is hypothesized that alterations in thyroid function can lead to changes in POMC and thyrotropin-releasing hormone, indicating a bidirectional relationship where thyroid dysfunction could lead to RLS and vice versa.

## Conclusions

Our case underscores the imperative need for a thorough evaluation during the diagnosis of RLS, highlighting the significance of ruling out mimics and underlying conditions, with a particular emphasis on thyroid dysfunctions. It is important to consider thyroid dysfunctions when RLS is suspected.
